# Characterization of Parkinson’s disease using blood-based biomarkers: A multicohort proteomic analysis

**DOI:** 10.1371/journal.pmed.1002931

**Published:** 2019-10-11

**Authors:** Marijan Posavi, Maria Diaz-Ortiz, Benjamine Liu, Christine R. Swanson, R. Tyler Skrinak, Pilar Hernandez-Con, Defne A. Amado, Michelle Fullard, Jacqueline Rick, Andrew Siderowf, Daniel Weintraub, Leo McCluskey, John Q. Trojanowski, Richard B. Dewey, Xuemei Huang, Alice S. Chen-Plotkin

**Affiliations:** 1 Department of Neurology, Perelman School of Medicine at the University of Pennsylvania, Philadelphia, Pennsylvania, United States of America; 2 National Institute of Neurological Disease and Stroke, National Institutes of Health, Bethesda, Maryland, United States of America; 3 Department of Psychiatry, Perelman School of Medicine at the University of Pennsylvania, Philadelphia, Pennsylvania, United States of America; 4 Department of Pathology and Laboratory Medicine, Perelman School of Medicine at the University of Pennsylvania, Philadelphia, Pennsylvania, United States of America; 5 Department of Neurology and Neurotherapeutics, Clinical Center for Movement Disorders at the University of Texas Southwestern Medical Center, Dallas, Texas, United States of America; 6 Department of Neurology, Penn State College of Medicine, Hershey, Pennsylvania, United States of America; University of Utah, UNITED STATES

## Abstract

**Background:**

Parkinson’s disease (PD) is a progressive neurodegenerative disease affecting about 5 million people worldwide with no disease-modifying therapies. We sought blood-based biomarkers in order to provide molecular characterization of individuals with PD for diagnostic confirmation and prediction of progression.

**Methods and findings:**

In 141 plasma samples (96 PD, 45 neurologically normal control [NC] individuals; 45.4% female, mean age 70.0 years) from a longitudinally followed Discovery Cohort based at the University of Pennsylvania (UPenn), we measured levels of 1,129 proteins using an aptamer-based platform. We modeled protein plasma concentration (log_10_ of relative fluorescence units [RFUs]) as the effect of treatment group (PD versus NC), age at plasma collection, sex, and the levodopa equivalent daily dose (LEDD), deriving first-pass candidate protein biomarkers based on *p*-value for PD versus NC. These candidate proteins were then ranked by Stability Selection. We confirmed findings from our Discovery Cohort in a Replication Cohort of 317 individuals (215 PD, 102 NC; 47.9% female, mean age 66.7 years) from the multisite, longitudinally followed National Institute of Neurological Disorders and Stroke Parkinson’s Disease Biomarker Program (PDBP) Cohort. Analytical approach in the Replication Cohort mirrored the approach in the Discovery Cohort: each protein plasma concentration (log_10_ of RFU) was modeled as the effect of group (PD versus NC), age at plasma collection, sex, clinical site, and batch. Of the top 10 proteins from the Discovery Cohort ranked by Stability Selection, four associations were replicated in the Replication Cohort. These blood-based biomarkers were bone sialoprotein (BSP, Discovery false discovery rate [FDR]-corrected *p* = 2.82 × 10^−2^, Replication FDR-corrected *p* = 1.03 × 10^−4^), osteomodulin (OMD, Discovery FDR-corrected *p* = 2.14 × 10^−2^, Replication FDR-corrected *p* = 9.14 × 10^−5^), aminoacylase-1 (ACY1, Discovery FDR-corrected *p* = 1.86 × 10^−3^, Replication FDR-corrected *p* = 2.18 × 10^−2^), and growth hormone receptor (GHR, Discovery FDR-corrected *p* = 3.49 × 10^−4^, Replication FDR-corrected *p* = 2.97 × 10^−3^). Measures of these proteins were not significantly affected by differences in sample handling, and they did not change comparing plasma samples from 10 PD participants sampled both on versus off dopaminergic medication. Plasma measures of OMD, ACY1, and GHR differed in PD versus NC but did not differ between individuals with amyotrophic lateral sclerosis (ALS, *n* = 59) versus NC. In the Discovery Cohort, individuals with baseline levels of GHR and ACY1 in the lowest tertile were more likely to progress to mild cognitive impairment (MCI) or dementia in Cox proportional hazards analyses adjusting for age, sex, and disease duration (hazard ratio [HR] 2.27 [95% CI 1.04–5.0, *p* = 0.04] for GHR, and HR 3.0 [95% CI 1.24–7.0, *p* = 0.014] for ACY1). GHR’s association with cognitive decline was confirmed in the Replication Cohort (HR 3.6 [95% CI 1.20–11.1, *p* = 0.02]). The main limitations of this study were its reliance on the aptamer-based platform for protein measurement and limited follow-up time available for some cohorts.

**Conclusions:**

In this study, we found that the blood-based biomarkers BSP, OMD, ACY1, and GHR robustly associated with PD across multiple clinical sites. Our findings suggest that biomarkers based on a peripheral blood sample may be developed for both disease characterization and prediction of future disease progression in PD.

## Abstract 2

Alice Chen-Plotkin and colleagues investigate possible biomarkers for diagnosis and progression of Parkinson’s disease.

## Author summary

**Why was this study done?:**

- No blood tests currently exist that distinguish people with Parkinson’s disease (PD) from neurologically normal individuals or that predict the rate of disease progression in people who have already been diagnosed with PD.
- Blood tests that distinguish people with PD would be helpful for confirmation of diagnosis (diagnostic biomarkers), whereas blood tests that predict the rate of disease progression (prognostic biomarkers) would be helpful for clinical trials and clinical care.

**What did the researchers do and find?:**

- We screened more than 1,000 blood-based proteins from 527 people with PD, amyotrophic lateral sclerosis (ALS), or neither neurological disease in order to discover new diagnostic and prognostic biomarkers. We used one group of participants to identify potential biomarkers and then used a separate group of participants to confirm these biomarkers.
- We found that blood levels of four proteins—bone sialoprotein (BSP), osteomodulin (OMD), aminoacylase-1 (ACY1), and growth hormone receptor (GHR)—consistently differed in people with PD compared to people without PD.
- We found that lower GHR levels at baseline predicted a faster rate of cognitive decline in people with PD.

**What do these findings mean?:**

- Levels of some blood proteins consistently differ between people with versus without PD, and some of these proteins also predict which PD individuals may have faster progression of disease.
- It may be possible to develop blood-based tests to help confirm PD diagnosis and predict disease progression.

## Introduction

Parkinson’s disease (PD) is characterized by progressive loss of dopaminergic neurons in the substantia nigra, resulting in a clinical syndrome defined by bradykinesia, rigidity, tremor, and postural instability [1]. By the time a clinical diagnosis is made, 50% of nigral dopaminergic neurons may already be lost [2], suggesting a long prodromal phase during which intervention may be possible. Current medical practice for the diagnosis of PD relies almost entirely on clinical examination, with no laboratory-based testing available. Although a United States Food and Drug Administration (FDA)-approved, radioligand-based dopamine transporter imaging test (DaTSCAN) can confirm degeneration of dopaminergic neurons [3], time and expense have prevented its widespread adoption in clinical settings, and a positive result is not diagnostic for PD, because other degenerative neurologic diseases such as multiple systems atrophy exhibit similar findings. Moreover, even within PD, considerable heterogeneity in clinical presentation exists, with highly variable rates of both cognitive and motor progression over time [4]. At present, no clinical or research-based tests exist to predict PD disease progression, despite widespread recognition that such predictive tools are vital to the field [5]. Thus, the advent of blood-based markers to molecularly define PD individuals and to predict longitudinal progression in PD could transform clinical practice and the development of disease-modifying therapies.

To date, biomarker studies in PD have largely focused on candidate approaches, with an emphasis on protein measures obtained in the cerebrospinal fluid (CSF) [6,7], which is considerably more difficult to obtain than blood in a busy clinical setting. Although a handful of biomarkers nominated using these candidate approaches consistently differ comparing PD and control individuals (e.g., CSF measures of total alpha-synuclein [8]), individual marker effect sizes are small, and the scarcity of robust biomarkers limits the ability to develop multimarker panels for better discriminatory power. Moreover, the field largely lacks protein biomarkers that predict cognitive or motor progression across multiple cohorts [7]. Thus, we aimed to discover novel blood-based biomarkers for differentiation of individuals with PD from control individuals, as well as prediction of rate of PD progression. We approached this problem by screening >1,000 plasma proteins using an aptamer-based platform [9] in a discovery–replication design.

## Methods

**Fig 1:**
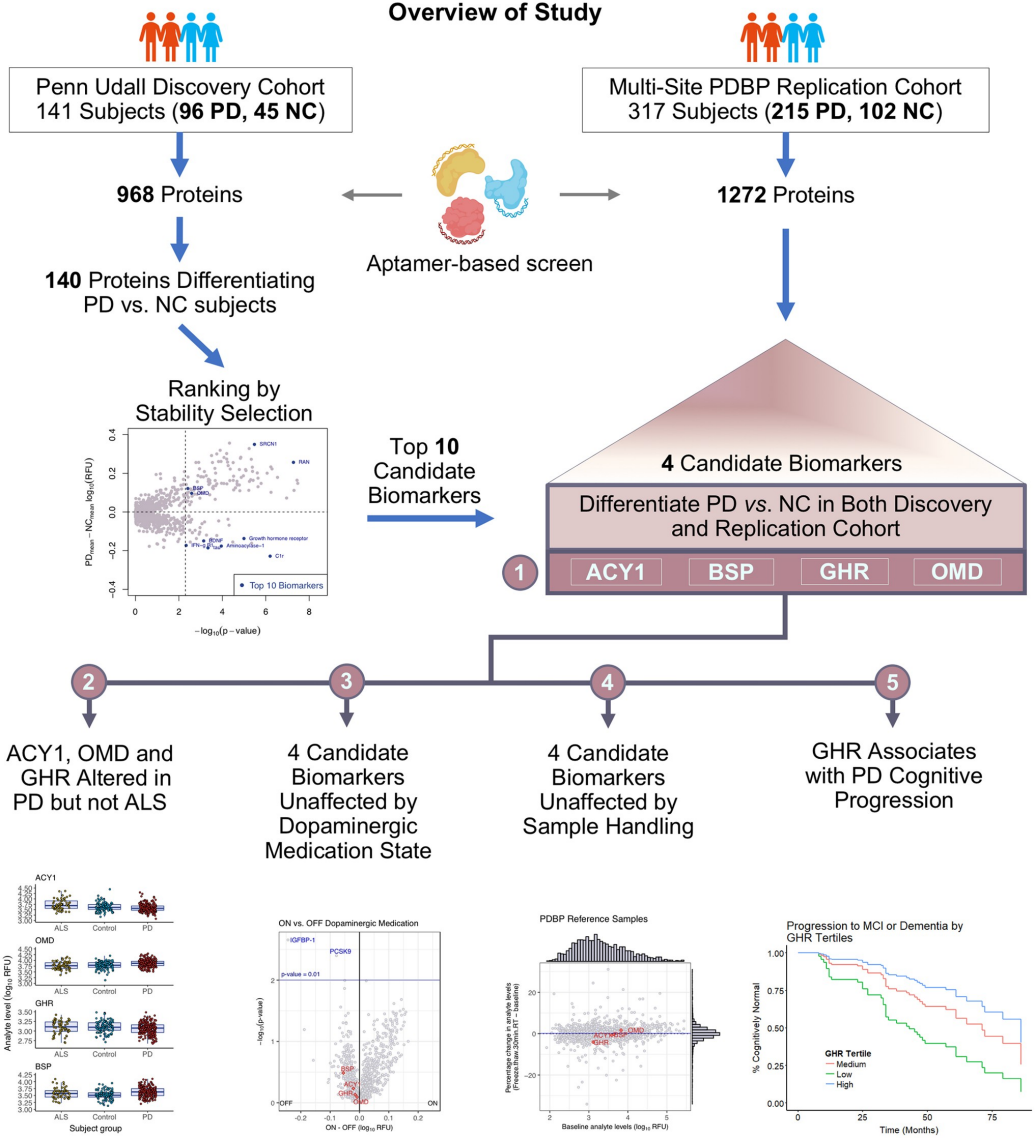
Overview of study. We used SOMAScan 1.1k (left panel) and 1.3k (right panel) assays to quantify plasma protein levels in the Discovery and Replication Cohorts, respectively. Multiple linear regression models revealed 140 proteins that differed in PD versus NC individuals in the Discovery Cohort (*p <* 0.005). Stability Selection with LASSO was then used to rank the 140 candidate proteins from the Discovery Cohort, obtaining the top 10 most robust and stable proteins. Four (ACY1, BSP, GHR, OMD) of these 10 proteins differed in PD versus NC samples in the Discovery Cohort and also differentiated PD versus NC in the Replication Cohort (middle panel, 1). These were tested for specificity for PD (2), influence of dopaminergic medication or sample handling (3, 4), and ability to predict subsequent rates of cognitive decline (5). ACY1, aminoacylase-1; ALS, amyotrophic lateral sclerosis; BSP, bone sialoprotein; GHR, growth hormone receptor; LASSO, least absolute shrinkage and selection operator; MCI, mild cognitive impairment; NC, neurologically normal control; OMD, osteomodulin; PD, Parkinson’s disease; PDBP, Parkinson’s Disease Biomarker Program.

To identify biomarkers that might characterize individuals with PD, 1,129 (Discovery Cohort) and 1,305 (Parkinson’s Disease Biomarker Program [PDBP] Replication Cohort) plasma proteins were screened from 527 individuals, and their clinical data were analyzed (Fig 1). The clinical data and plasma samples were acquired from the University of Pennsylvania (UPenn) Udall Cohort (Discovery Cohort) and from a multisite PDBP Replication Cohort (Table 1). The study consisted of three major steps: (1) differentiation of PD from neurologically normal control (NC) participants (96 PD, 45 NC) in a single-site Discovery Cohort, using levels of 968 proteins that passed quality control (QC) metrics, obtained by an aptamer-based platform assay; (2) independent replication of the top biomarker candidates from step 1 in a multisite Replication Cohort (215 PD, 102 NC participants); and (3) prediction of PD progression using the biomarker candidates that replicated across the single-site Discovery Cohort and multisite Replication Cohort. These steps are summarized in Fig 1, parts of which were created with Biorender.com. The data were normalized, processed, and analyzed using the statistical software R [10]. This study is reported as per the Strengthening the Reporting of Observations Studies in Epidemiology (STROBE) guidelines (S1 STROBE Checklist). At study outset, the analysis plan (S1 Analysis Plan) was to flexibly investigate the Discovery Cohort and then perform an analysis mirroring the Discovery Cohort analysis in the Replication Cohort, with the two additional covariates of clinical site and batch, if site or batch effects were observed in the transition from a single-site/single-batch discovery phase to a multisite/multibatch replication phase. The ultimate analysis plan only differed from the predetermined plan in that the levodopa equivalent daily dose (LEDD) was not included as a covariate in the Replication Cohort when we found that not all PDBP PD participants had available LEDD data. Detailed methods are described below.

**Table 1:** Demographic characteristics of study participants.

	Penn Udall Discovery Cohort		PDBP Replication Cohort (Penn State and UTSW)		Penn ALS	BioFIND	
Disease group	PD	NC	PD	NC	ALS*	ON	OFF
Participants total	96	45	215	102	59	10	
Female/Male	42/54	22/23	101/114	51/51	26/33	5/5	
*p*-Value	0.59^a^		0.76^c^			N/A	
Age at plasma sampling mean ± SD (years)	69.9 ± 7.52	70.2 ± 10.04	66.8 ± 8.6	66.1 ± 10.5	61.9 ± 10.5	73.5 ± 6.13	
*p*-Value	0.65^b^		0.84^e^ (PD versus NC)			N/A	
			0.02^e^ (ALS versus NC); 0.002^e^ (ALS versus PD)				
Collection site number	N/A	N/A	215	102	N/A	N/A	
Penn State/UTSW			100/115	78/24			
*p*-Value	N/A	N/A	< 0.01^a^		N/A	N/A	
Blood samples storage at −80 °C mean ± SD (years)	1.44 ± 0.88	1.43 ± 1.53	3.62 ± 0.54	3.46 ± 0.72	5.15 ± 2.01	3.26 ± 0.76	3.24 ± 0.76
p-value	0.977^d^		0.33^e^ (PD versus NC)			0.943^d^	
			<0.001^e^ (ALS versus PD and ALS versus NC)				
Somalogic plate number	N/A	N/A	215	102	N/A	N/A	
Batch 1/2/3/4/5			49/49/48/48/21	21/20/24/24/13			
*p*-Value	N/A	N/A	0.88^c^		N/A	N/A	
Baseline DRS (UPenn) or MoCA (UTSW) mean ± SD	136.95 ± 9.96	N/A	26.47 ± 2.58^‡^	N/A		N/A	

^a^Fisher exact test.

^b^Mann-Whitney test.

^c^Chi-squared test.

^d^*t* test.

^e^Tukey HSD test.

^‡^ For 110 UTSW participants with PD considered for longitudinal analyses.

Abbreviations: ALS, amyotrophic lateral sclerosis; DRS, Mattis Dementia Rating Scale-2; HSD, honest significant difference; MoCA, Montreal Cognitive Assessment; NC, neurologically normal control; PD, Parkinson’s disease; PDBP, Parkinson’s Disease Biomarker Program; SD, standard deviation; UPenn, University of Pennsylvania; UTSW, University of Texas Southwestern Medical Center

### Cohorts and sample collection

#### UPenn Udall Discovery Cohort

During the period between 2013 and 2015, blood plasma samples and clinical data were collected from 97 PD individuals and 45 NCs enrolled to participate in research approved by the UPenn Institutional Review Board (IRB). All PD individuals met the diagnostic criteria of the United Kingdom Parkinson’s Disease Brain Bank [9] and were part of a longitudinal, extensively characterized cohort at UPenn [11]. In order to control for environmental biases, we ensured that PD and control groups did not differ by age or sex, and NCs were recruited primarily from the unaffected spouses of PD individuals from the same clinic. Samples were acquired according to IRB-approved protocols as previously described by Chen-Plotkin and colleagues [12]. Written informed consent was obtained at study enrollment. One PD sample had outlier values in preprocessing and normalization steps on the aptamer-based assay and was excluded from further analyses. A total of 96/97 PD and all NC samples passed our QC criteria (see Preprocessing and QC of SOMAScan protein data for description of preprocessing and normalization) and were included in subsequent analyses.

#### Multisite PDBP Replication Cohort

Results were tested in a Replication Cohort in order to account for possible environmental and technical biases in our analysis. The Replication Cohort blood samples (collected in the period between 2013 and 2015) and clinical data were obtained from the PDBP Cohort [13], originating from research participants seen at two PDBP sites: Penn State University (Penn State, 100 PD and 78 NC) and the University of Texas Southwestern Medical Center (UTSW, 115 PD and 24 NC). One PD participant from UTSW and two NC participants from Penn State were excluded from analyses because of outlier measurements for a high proportion of SOMAScan proteins. Longitudinal follow-up with cognitive testing by the Montreal Cognitive Assessment (MoCA) was additionally obtained, and associations with biomarker levels analyzed, for UTSW PD participants. Each PDBP Center’s local IRB approved study protocols, and all participants were consented for the study.

#### Ethics statement for human participant research

For the Discovery Cohort, the IRB of UPenn approved the human participant research in this study. Written informed consent was obtained from Discovery Cohort participants. For the Replication Cohort, each PDBP Center’s local IRB approved study protocols, and all participants provided written informed consent for participation in PDBP. As one goal of the PDBP is to provide a biorepository of samples from a well-characterized set of individuals, participants consented to sharing of samples and deidentified data with investigators approved by the Biospecimen Review Access Committee at the time of enrollment.

#### Pooled reference samples

To investigate the effect of plasma handling on detected biomarker level, multiple identical aliquots of pooled plasma samples from the Discovery (UPenn reference pool) and Replication Cohorts (PDBP reference pool) were used. The UPenn reference pool was prepared by mixing samples from 450 PD and NC individuals. The PDBP reference pool included plasma samples from 13 PD and NC individuals. For these studies, one aliquot was assayed by SOMAScan directly, whereas an identical aliquot was subjected to incubation at room temperature for 30 minutes, followed by an extra freeze–thaw cycle, before assaying by SOMAScan.

#### BioFIND Cohort samples

The effect of levodopa therapy on plasma protein concentration was tested on 10 randomly selected PD individuals (5 females and 5 males above 50 years of age) from the BioFIND Cohort (Table 1) [14]. Plasma samples from the BioFIND Cohort were collected at two different visits: at baseline (when PD individuals had samples collected while taking their usual dopaminergic medication—i.e., ON medication) and 2 weeks after the baseline visit (when PD individuals had samples collected after an overnight washout of dopaminergic medication—i.e., OFF medication), as described by Kang and colleagues [14]. All study protocols and recruitment strategies for BioFIND were approved by the IRBs for the University of Rochester Clinical Trials Coordination Center (CTCC) and individual clinical sites.

### Protein quantification

Samples from the Discovery and Replication Cohorts were assayed using the 1.1k and 1.3k Assay versions of the SOMAScan platform (Somalogic, Boulder, CO, USA [9]) in two separate runs, with operators blinded to disease status. This platform is based on protein-capture slow off-rate modified aptamers (SOMAmers), which are chemically modified oligonucleotides with specific affinity to recombinant protein targets, developed by in vitro selection (SELEX) as previously described [15,16].

The specific steps of the SOMAScan assay have been described in detail in prior publications [9,17,18], as well as technical white papers at www.somalogic.com. In brief, plasma samples were incubated with reagent mixes containing SOMAmers to allow for equilibrium binding of fluorophore-tagged aptamers to their protein targets. Next, a series of partitioning and washing steps were used to capture only SOMAmers that were bound to their cognate proteins. Finally, the protein-bound oligonucleotides were released from the protein complex, captured by complementarity, and quantified using DNA hybridization arrays.

To adjust for technical biases, the hybridization arrays were normalized and calibrated using data from a reference set of pooled plasma samples that was run with each batch. Raw Somalogic data in relative fluorescence units (RFUs) were log_10_-transformed prior to analysis. A total of 142 plasma samples (97 PD and 45 NC) from the Discovery Cohort were assayed for 1,129 proteins (1.1k Assay), and 320 plasma samples (216 PD and 104 NC) from the Replication Cohort were assayed for 1,305 proteins (1.3k Assay). The Replication Cohort samples were assayed in batches of 85, distributed in five different plates, along with plasma reference pool samples, 59 amyotrophic lateral sclerosis (ALS) samples from the UPenn biorepository, and 20 samples from the BioFIND biorepository.

### Preprocessing and QC of SOMAScan protein data

Plasma samples from the Discovery and Replication Cohorts were assayed in separate Somalogic runs. Discovery Cohort plasma samples were analyzed, along with 13 plasma calibrator samples, hybridization controls, and two buffer control samples. The reference pool samples (*n* = 8), Penn Udall ALS samples (*n* = 59), and BioFIND samples (*n* = 20 from 10 individuals with PD) were assayed along with plasma samples from the Replication Cohort.

Run QC standards were derived from metrics obtained during assay development, and preprocessing and normalization methods are described in detail in a technical white paper [19]. In brief, sample data were first normalized to eliminate hybridization artifacts, using “spiked in” hybridization controls. Median normalization was subsequently applied for each sample to remove other intraplate biases. For the SOMAScan assay, the hybridization control and median scale factors are expected to be in the range of 0.4–2.5 (±1.32 on log_2_ scale). All samples had hybridization scale factors in the acceptable range, except one PD sample from the Discovery Cohort, which was excluded from downstream analyses.

In the next step, two QC criteria were implemented to filter SOMAScan protein data. The overall intraplate technical variability of SOMAScan assay was assessed by using three QC samples (identical aliquots from three different reference sample pools) run in triplicate (for a total of nine samples). These three sets of triplicates were placed randomly within the batches of biological samples in order to capture intraplate variability. The coefficients of variation (CVs) from these three sets of triplicate QC samples were calculated (i.e., for each protein, three CVs were calculated) using the raw Somalogic data (in RFUs). Proteins showing CVs greater than 0.2 from any one of the triplicates were excluded from downstream analyses. There were 36 and 33 proteins in the Discovery and Replication Cohorts, respectively, with CV > 0.2 in at least one of three runs (S1 Fig). The second filter, which involved removing proteins with >25% measurements outside of the lower limit of detection (LLOD) or upper limit of detection (ULOD), was applied to the Discovery Cohort only, as limits of detection were not provided for subsequent versions of the SOMAScan. This resulted in elimination of an additional 125 proteins (S1A Fig), leaving a total of 968 Discovery Cohort proteins for downstream analyses.

### Data processing and cross-sectional statistical analyses

#### Nomination of proteins that differed in PD versus NC group

To detect proteins whose plasma concentration associated significantly with disease category (PD versus NC), multiple linear regression models were employed. In the Discovery Cohort, each protein plasma concentration (log_10_ of RFU) was modeled as the effect of treatment group (PD versus NC), age at plasma collection, sex, and the LEDD. A total of 140 candidate biomarkers with *p*-value of group effect < 0.005 (PD versus NC) were nominated (S1 Table) for downstream analyses (hierarchical clustering and Stability Selection) from the Discovery Cohort. False discovery rate (FDR)-corrected *p*-values were also derived for these biomarkers using the Benjamini-Hochberg method [20].

#### Hierarchical clustering and heatmap generation

A heatmap was generated using the function heatmap.2 from the R package gplots [21]. Raw Somalogic data (RFUs) were log-transformed and then centered and scaled. Both participants and proteins were hierarchically clustered by euclidean distance and average linkage using the hclust function [10].

#### Stability selection ranking

We performed Stability Selection [22] (variable selection based on subsampling in combination with least absolute shrinkage and selection operator [LASSO] [23]) on Discovery Cohort data (96 PD, 45 NC). To rank candidate biomarkers, the R BioMark package across 100,000 jackknifed iterations [24] was employed. At each iteration, 30% of the proteins and 10% of the samples were left out of the bag, and LASSO was used to feature-select for variables on the remaining data. The proportion of iterations in which LASSO reported a nonzero coefficient was used to rank the proteins, generating a list of the top 10 proteins for evaluation in the Replication Cohort.

#### Replication Cohort analyses

In the Replication Cohort, each protein plasma concentration (log_10_ of RFU) was modeled as the effect of group (PD versus NC), age at plasma collection, sex, clinical site (UTSW versus Penn State), and batch (five plates). LEDD was not included as a covariate in the Replication Cohort when we found that not all PDBP PD participants had available LEDD data in the PDBP Data Management Resource. Analyses focused on the top 10 proteins from the Discovery Cohort, as ranked by Stability Selection; *p*-values were corrected for multiple hypothesis testing using the Benjamini-Hochberg method [20]. For the four validated proteins, a similar analysis was repeated including 59 ALS participants. Protein plasma concentration (log_10_ of RFU) was modeled as the effect of disease group (NC, PD, or ALS), age at plasma collection, sex, and clinical site. Disease group coefficients were extracted, and *p*-values were adjusted using the Benjamini-Hochberg method.

#### Testing the effects of levodopa therapy

To test the effect of levodopa therapy on plasma protein levels (log_10_ of RFU), a paired *t* test was applied to each of the proteins assayed. Nominal (unadjusted) paired *t* test *p*-values are presented in S5 Table. In addition, analyses were repeated, and *p*-values were obtained by paired permutation testing, which avoids assumptions of normality. Results were unchanged (S2 Fig).

#### Associations with cognition

Spearman’s rank-order correlation was calculated for baseline Mattis Dementia Rating Scale-2 (DRS) and biomarker (log_10_ of RFU) levels using the R function cor.test [10]. *p*-Values were adjusted for FDR by the Benjamini-Hochberg method using the R function p.adjust [10].

### PD progression analysis

#### Linear mixed-effects model analysis

Linear mixed-effects models were fitted to determine the effect of biomarker level on the rate of cognitive decline using the R package nlme [25]. In the Discovery Cohort, only participants with DRS scores measured within 6 months of the blood draw as well as at least one subsequent DRS score were included (*n* = 91), for an average follow-up period of 3.5 years. Our model incorporated DRS as the response variable, with age, sex, disease duration, baseline DRS, and the time-by-protein interaction as fixed effects and participant as a random effect. The same analysis was repeated adjusting for years of education as an additional fixed effect. The time-by-protein interaction coefficients were extracted, and the *p*-values for the interaction term were adjusted for FDR by the Benjamini-Hochberg method using the R function p.adjust [10].

#### Survival analysis

In both the Discovery and Replication Cohorts, individuals were divided into low-, medium-, or high-biomarker groups based on (log_10_ of RFU) biomarker tertiles. For the Discovery Cohort, we extracted cognitive diagnoses based on clinical consensus diagnosis as previously described [11], and PD individuals with either a cognitive diagnosis of dementia at baseline or a diagnosis coded as normal following a baseline diagnosis of mild cognitive impairment (MCI) were excluded from the analysis, leaving 86 individuals for survival analysis. Events were defined as conversion from normal to MCI, normal to dementia, or MCI to dementia, for a total of 38 events. For the Replication Cohort, cognitive categorization was based on published MoCA norms (MoCA 26–30 = normal, MoCA 21–25 = MCI, and MoCA 20 or less = dementia) [26], and events were defined and participants with PD filtered in the same way as for the Discovery Cohort, for a total of 26 events observed in 74 individuals (followed for an average of 2.8 years). Survival analysis was carried out in R using the survival [27] package. Cox proportional hazards analyses were performed using the function coxph [27] to test whether biomarker tertile groups have an effect on the likelihood of an event, adjusting for age, sex, and disease duration. Analyses were repeated including years of education as an additional covariate. Results were visualized as Cox regression–adjusted curves or forest plots using the ggadjustedcurves and ggforest functions from the R survminer package [28]. Models with and without education were compared using the anova.coxph function from the R survival package [27].

## Results

### Discovery screen for plasma proteins differentiating PD from NC

From the original set of 1,129 proteins assayed in the single-site Discovery Cohort, 968 (85.7%) met QC standards (S1 Fig and S1 Table). These 968 proteins were retained for downstream analyses.

Proteins differentiating PD from NC samples in the Discovery Cohort were nominated using a linear model associating concentration of each of these 968 proteins with disease state, adjusting for LEDD [29], age at plasma draw, and sex, generating an initial candidate list of 140 biomarkers associated at a nominal *p*-value < 0.005 with PD; correction for multiple hypothesis testing using the Benjamini-Hochberg method [20] demonstrated that all 140 proteins met the additional criterion of associating with disease state with an FDR-corrected *p*-value < 0.05 (S2 Table).

**Fig 2:**
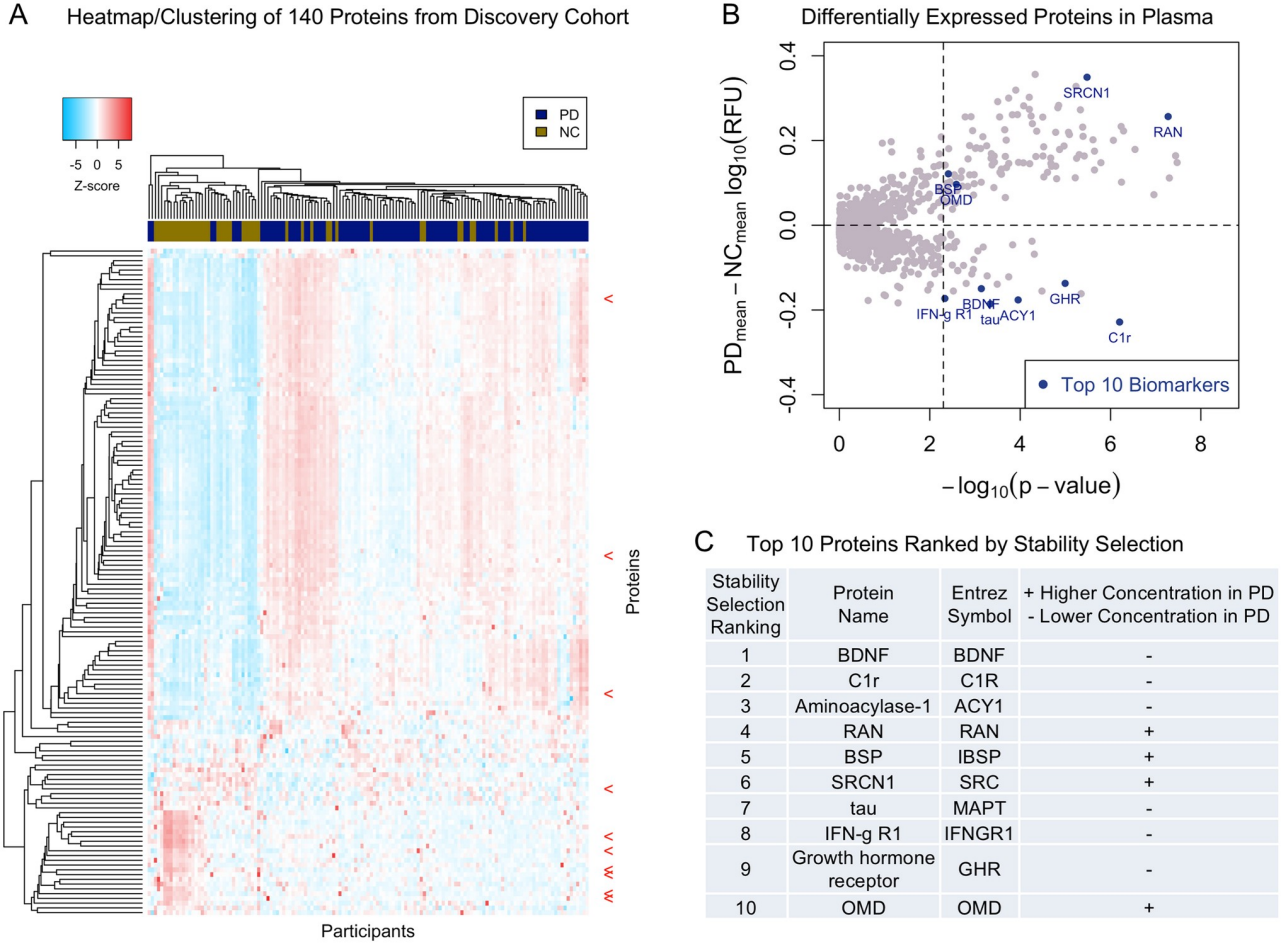
Identification of proteins differentiating PD and NC samples in the Discovery Cohort. (A) Heatmap showing the plasma levels of 140 candidate biomarkers differentially expressed (*p <* 0.005) between PD (*n* = 96) and NC (*n* = 45) in the Discovery Cohort (S2 Table). Blue color indicates lower and red color indicates higher protein expression, with intensities signifying magnitude of the change. Dendrogram clustering on the x-axis shows participant similarity (PD = navy, NC = gold), whereas clustering on the y-axis groups proteins according to similarity in their expression profiles. Red arrows indicate top 10 protein biomarkers from the Discovery Cohort. (B) Plot of differentially expressed proteins between PD and NC group in the Discovery Cohort. The x-axis corresponds to the significance (−log_10_ *p*-value) of the difference in protein levels between PD and NC group, and the y-axis displays the group mean difference between PD and NC (PD_mean_ log_10_ RFU − NC_mean_ log_10_ RFU). The top 10 proteins ranked by Stability Selection are labeled in blue. The vertical dotted line represents the *p*-value of 0.005. (C) Top 10 proteins as ranked by Stability Selection in the Discovery Cohort. BSP, bone sialoprotein; PD, Parkinson’s disease; NC, neurologically normal control; OMD, osteomodulin; RFU, relative fluorescence unit.

We next performed hierarchical clustering on these candidate markers to evaluate the correlation structure between groups of proteins and disease state. Unsupervised clustering revealed colinearity among subsets of these proteins, suggesting redundancies and possible shared relationships among many candidate biomarkers (Fig 2A). We thus employed Stability Selection [22], a meta-statistical tool that identifies consistently important features by repeated subsampling of the data, in order to identify the most robust, stable, and sparse set of discriminatory proteins; we ranked candidate biomarkers using the LASSO method across 100,000 jackknifed iterations [23,24]. The top 10 proteins from the Discovery Cohort ranked by Stability Selection, shown in Fig 2B and 2C, were advanced for replication.

### Replication of biomarker associations with PD in the PDBP Cohort

We next tested our top 10 stability-ranked markers for robustness in a separate Replication Cohort of 215 PD and 102 NC participants drawn from the multicenter PDBP [13] cohort (Table 1 and S3 Table). Analytical methods were identical to those used in the Discovery Cohort except that the Replication Cohort analysis additionally included clinical site and batch as covariates and did not include LEDD as a covariate, since LEDDs were not universally available for PDBP participants.

**Fig 3:**
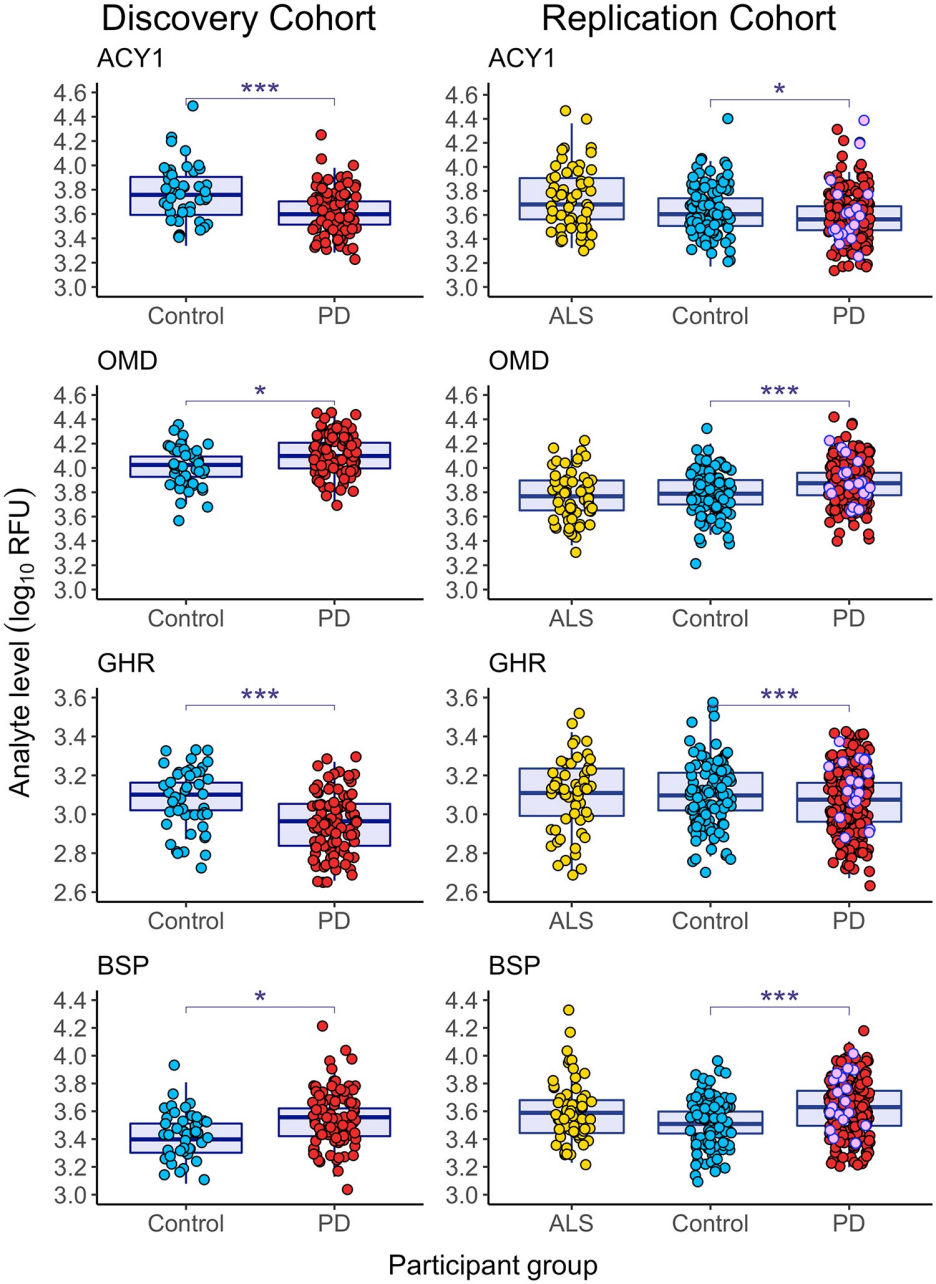
Blood-based biomarkers found in both Discovery and Replication Cohorts. Boxplots (median and IQR) showing the distribution of the top four biomarker protein levels (log_10_ of RFU) found in both the Discovery and Replication Cohorts according to diagnosis. The y-axis represents plasma protein levels (log_10_ of RFU), and the x-axis displays the diagnostic group, with each dot representing one research participant. In the Replication Cohort, 18 PD participants had never been treated with dopaminergic medication (pink dots). Biomarker measures for these never-treated PD participants did not differ from biomarker measures for PD participants treated with dopaminergic medication (Wilcoxon test nominal *p >* 0.05). FDR-adjusted (Benjamini-Hochberg method) *p < 0.05, ***p < 0.005. ACY1, aminoacylase-1; ALS, amyotrophic lateral sclerosis; BSP, bone sialoprotein; FDR, false discovery rate; GHR, growth hormone receptor; OMD, osteomodulin; PD, Parkinson’s disease; NC, neurologically normal control; RFU, relative fluorescence unit.

Despite the inevitable introduction of variability from a multisite, multibatch Replication Cohort, with slight differences in clinical data availability, four of the top 10 proteins that differed in PD versus NC samples in the Discovery Cohort also differed between PD and NC, with the same direction of effect, in the PDBP Replication Cohort (Fig 3). These protein biomarkers were bone sialoprotein (BSP, Discovery FDR-corrected *p* = 2.82 × 10^−2^, Replication FDR-corrected *p* = 1.03 × 10^−4^), osteomodulin (OMD, Discovery FDR-corrected *p* = 2.14 × 10^−2^, Replication FDR-corrected *p* = 9.14 × 10^−5^), aminoacylase-1 (ACY1, Discovery FDR-corrected *p* = 1.86 × 10^−3^, Replication FDR-corrected *p* = 2.18 × 10^−2^), and growth hormone receptor (GHR, Discovery FDR-corrected *p* = 3.49 × 10^−4^, Replication FDR-corrected *p* = 2.97 × 10^−3^, S4 Table).

### Biomarker measures in ALS, a neurodegenerative disease with motor and cognitive features

To determine whether each of these plasma proteins specifically characterize PD or whether they are seen across many neurodegenerative disease states, we additionally measured these proteins in 59 individuals with ALS (Table 1), a neurodegenerative disease that, like PD, has both motor and cognitive features. As shown in Fig 3 and corroborated by multiple linear regression adjusting for age, sex, and clinical site, with the exception of BSP, protein changes were seen in PD but not in ALS.

### Preanalytical variability and biomarker measures

**Fig 4:**
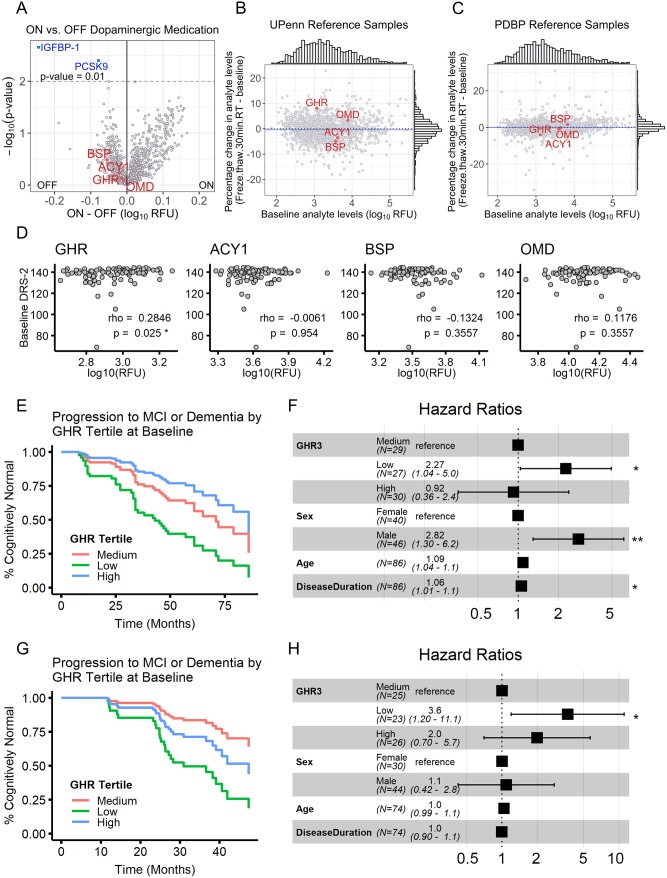
Top biomarkers are robust and predict cognitive trajectory. (A) Volcano plot showing the effect of dopaminergic therapy on plasma protein levels, tested in 10 PD individuals, comparing ON versus OFF medication state in the same individuals. Nominally significant differences in ON versus OFF state were found for only two proteins (paired *t* test nominal *p* < 0.01), and none of our four top biomarker proteins was affected substantially. (B-C) Effect of plasma handling (samples compared with versus without a 30-minute RT incubation followed by an extra freeze–thaw cycle) in two reference pools. Plasma handling caused substantial changes in levels of some proteins, but none of our four top biomarkers was affected. (D) Cross-sectional relationship between PD biomarker measures (log_10_ of RFU) and baseline cognitive function as measured by DRS score (Spearman’s correlation, *FDR-adjusted *p* < 0.05). (E-H) Cox proportional hazards analyses investigating differences in subsequent rates of clinical conversion to MCI or dementia, stratified by GHR measures at baseline in the Discovery Cohort (tertiles shown, panels E,F) and the Replication Cohort (tertiles shown, panels G,H). For the Replication Cohort, cognitive assignments were based on published norms for the MoCA, longitudinally assessed at the UTSW site. (E,G) Cox regression curves showing adjusted trajectories for each tertile of baseline GHR measures in each cohort. (F,H) Forest plots depicting hazard ratios for groups as defined by tertile of biomarker measures at baseline, sex, age, and disease duration. **p < 0.01. ACY1, aminoacylase-1; BSP, bone sialoprotein; DRS, Mattis Dementia Rating Scale-2; FDR, false discovery rate; GHR, growth hormone receptor; IGFBP-1, insulin-like growth factor binding protein 1; MCI, mild cognitive impairment; MoCA, Montreal Cognitive Assessment; OMD, osteomodulin; PCSK9, proprotein convertase subtilisin/kexin type 9; PD, Parkinson’s disease; PDBP, Parkinson’s Disease Biomarker Program; RFU, relative fluorescence unit; RT, room temperature; UPenn, University of Pennsylvania; UTSW, University of Texas Southwestern Medical Center.

Most individuals with PD are treated with dopaminergic medication, raising the concern that medication-based effects on the plasma proteome may be driving our biomarker signals. We addressed this concern in two ways. First, in our PDBP Replication Cohort, a subset of individuals with PD (*n* = 18) had never been treated with dopaminergic medications. We compared values of ACY1, OMD, GHR, and BSP in people with PD treated with dopaminergic medication versus those never treated with dopaminergic medications, and we found no significant differences (Wilcoxon test nominal *p*-value > 0.05 for all four proteins comparing PD treated versus not treated with dopaminergic medication, Fig 3). Second, we investigated samples from an additional multisite cohort—the BioFIND Study [14]—in which PD participants had blood drawn in two settings: (1) while on their customary dopaminergic medications and (2) after overnight washout of medication (S5 Table). Although some plasma proteins may be affected by medication state, none of our four biomarker proteins changed substantially when comparing ON medication and OFF medication states in the same individual (Fig 4A, S2 Fig).

To understand whether candidate protein biomarkers are robust to common sources of preanalytical variability, we investigated identical aliquots of pooled plasma samples from the Discovery Cohort (UPenn reference samples pool) and the PDBP Cohort (PDBP reference samples pool) that were subjected to differences in sample handling (with versus without 30 minutes at room temperature followed by an additional freeze–thaw of the sample). Whereas some proteins changed their levels by >30% based on differences in sample handling, none of our top proteins changed substantially in either pool (Fig 4B and 4C, S5 Table).

### GHR, ACY1, and OMD as predictors of cognitive decline

We next asked whether baseline levels of our candidate biomarkers predicted disease progression. Because cognitive symptoms are less affected by dopaminergic medication than motor symptoms, and because decline in cognition is variable but clinically important in PD [30], we investigated whether baseline levels of BSP, OMD, ACY1, or GHR predicted subsequent rates of cognitive decline.

In our extensively characterized Discovery Cohort participants, who have been followed for an average of 3.5 years after plasma sampling, cross-sectional analyses revealed minimal association between plasma biomarker levels and baseline cognitive scores on the multidomain DRS, which has been used extensively for cognitive assessment in PD [31] (Fig 4D). However, plasma levels of GHR, ACY1, and OMD predicted subsequent rates of cognitive change on the DRS in mixed-effects linear models adjusting for age, sex, disease duration, and baseline DRS score, with time-by-protein interaction coefficients of 0.0905 (GHR, FDR-corrected *p* = 8.72 × 10^−6^), 0.0478 (ACY1, FDR-corrected *p* = 2.574 × 10^−2^), and −0.0457 (OMD, FDR-corrected *p* = 2.574 × 10^−2^), respectively. Moreover, individuals with baseline levels of GHR and ACY1 in the lowest tertile were significantly more likely to clinically progress to MCI or dementia in Cox proportional hazards analyses adjusting for age, sex, and disease duration (hazard ratio [HR] 2.27 [95% CI 1.04–5.0, *p* = 0.04] for GHR [Fig 4E and 4F], and HR 3.0 [95% CI 1.24–7.0, *p* = 0.014] for ACY1 [S3 Fig]). Finally, correcting for education in our models did not affect our results (S6 Table, S3 Fig).

The PDBP Replication Cohort is less mature in follow-up than our Discovery Cohort. In addition, cognitive testing data are more limited, with variability among PDBP sites with respect to their collection of cognitive data and stage of PD. These limitations notwithstanding, scores on the MoCA were obtained at 6-month intervals for an average 2.8-year follow-up period for 74 PD individuals from the PDBP Replication Cohort, followed at UTSW. In these participants, we classified each individual as having normal cognition, MCI, or dementia for each time point according to published norms for the MoCA [26]. Using the same Cox proportional hazards models (i.e., adjusted for age, sex, and disease duration) as in the Discovery Cohort, we found that individuals with baseline levels of GHR in the lowest tertile were more likely to progress to MoCA scores in the MCI or dementia range (HR 3.6 [95% CI 1.20–11.1, *p* = 0.02]) in the Replication Cohort as well (Fig 4G and 4H). Moreover, just as in the Discovery Cohort, additional correction for education in our model did not affect results (S4 Fig).

## Discussion

In this study, we investigated multiple cohorts in a discovery–replication design to develop novel PD biomarkers, starting from an unbiased screen of approximately 1,000 plasma proteins. We found four top biomarker candidates—ACY1, BSP, GHR, and OMD—that replicated across a single-site Discovery Cohort and a multisite Replication Cohort, were robust to common sources of preanalytical variability, and did not differ in paired samples from PD participants on versus off dopaminergic medication. In analyses of longitudinal data, we showed that baseline levels of ACY1 and GHR—and, to a lesser extent, OMD—associated with subsequent rates of cognitive decline in our Discovery Cohort, with baseline GHR predicting subsequent cognitive course in the Replication Cohort as well.

The PD biomarkers found here have not, to our knowledge, been previously reported in the neurodegenerative disease literature. However, unbiased screens—most commonly exemplified by the genome-wide association study in human genetics—often yield unexpected new directions for investigation [6]. We note, however, that GHR and insulin-like growth factor (IGF-1, a well-known effector produced in response to growth hormone [GH]-GHR signaling), are expressed in the brain [32,33] and have been implicated in both physiological and pathological events in the brain. GH-GHR-IGF-1 signaling has been implicated in neural stem cell differentiation and proliferation during embryonic development [33–35], adult neurogenesis in rodents [36,37], age-related cognitive decline [38,39], and neuroprotection against neurological insults such as hypoxic-ischemic injury [40,41], pointing toward potential links between this pathway and protection from neurodegeneration. Future studies using mendelian randomization techniques [42] or manipulation of biomarker levels in model systems are needed, however, to truly elucidate potential mechanisms leading to the biomarker signatures described here.

Strengths of this study include attention to reproducibility, as well as consideration of real-world factors that influence downstream translational potential. With respect to reproducibility, we highlight four aspects. First, the ranking of top candidate proteins from the Discovery Cohort by Stability Selection, rather than strict ordering by *p*-value, guards against concerns regarding overfitting. Second, biomarkers described here had FDR-corrected *p*-values < 0.05 in both the single-site Discovery and multisite PDBP Replication Cohorts, attesting to the robustness of our findings. Third, the analysis strategy in the Replication Cohort was prespecified to mirror that of the Discovery Cohort, with only two differences: (1) the inclusion of site and batch as additional covariates, justified by the move from single-site/single-batch to multisite/multibatch phases of analysis, and (2) the removal of LEDD as a covariate, necessitated by lack of these data uniformly across all Replication Cohort participants. Fourth, we note that baseline levels of GHR, ACY1, and, to a lesser extent, OMD predicted future cognitive decline in individuals with PD from the Discovery Cohort. Moreover, despite differences in cognitive scale and clinical site used, as well as stage of PD assessed (all factors known to affect measures of cognition over time), lower levels of GHR also predicted faster cognitive decline in the Replication Cohort. Aside from meeting a clear need for biomarkers predicting PD progression [7], the association of the same proteins with disease class as well as disease progression increases confidence in these biomarker candidates, since the gradation of levels within PD according to one measure of pathophysiological severity (rate of cognitive decline) suggests that the differences between PD and NC are not due to a hidden confounding variable differentiating these two groups. With respect to downstream translational considerations, we investigated aspects of real-world variability, demonstrating that all four top biomarkers reported here are not substantially affected by dopaminergic medication state or common sources of noise related to sample handling. We also emphasize the fact that our biomarker candidates are measured from the blood plasma, allowing for collection in any routine phlebotomy setting.

Our study also has limitations. First, our study relies on an aptamer-based platform [9] for plasma protein measures. Although this is a powerful approach for large-scale screening, downstream translation will likely require development of alternative protein assays that (1) yield absolute protein quantities rather than the RFUs analyzed here and (2) confirm assay specificity. Second, although we have adjusted for dopaminergic medication effects where possible and directly analyzed the effect of dopaminergic medication state on protein measures, our study cannot rule out small effects of dopaminergic medication on candidate protein measures, since overnight washout of dopaminergic medication does not fully mitigate medication effects. Thus, evaluation of candidate protein biomarkers in unmedicated early symptomatic or even presymptomatic, high-risk cohorts is a fruitful future avenue. Third, although we have demonstrated that plasma levels of ACY1, GHR, and OMD are to some extent specific to PD, in that they are not similarly changed in ALS, it is still possible that some of these protein biomarkers may show similar changes in other neurodegenerative diseases that were not tested. We note, however, that increasing appreciation for the overlap of pathology across various neurodegenerative diseases—individuals with PD, for example, are highly likely to have concomitant Alzheimer’s disease (AD) neuropathology at autopsy [43]—suggests that overlap in biomarkers across current clinical categories may reflect overlap in pathophysiological mechanism, rather than a poor biomarker. Fourth, for our longitudinal analyses, we assessed cognitive change in order to understand whether candidate biomarkers predicted disease progression. We chose to investigate cognitive decline both because of the major morbidity associated with this aspect of disease progression and because cognition is not as affected by dopaminergic medication as motor performance. Because the majority of PD participants studied here were assessed while taking dopaminergic medication, motor performance would be expected to reflect not only underlying disease state over time (what we aim to measure) but also medication response and timing of most recent dose of medication, adding considerable noise. Thus, although our study found that several of these candidate biomarkers may predict disease progression along one axis (cognitive decline), whether they also predict motor progression is an open question—one that might also be answered by future study in early symptomatic PD individuals not yet taking dopaminergic medication.

In summary, we present our findings from unbiased screening of >1,000 plasma proteins in multiple PD cohorts (a single-site Discovery Cohort, the multicenter PDBP Replication Cohort, and the multicenter BioFIND Cohort), as well as disease and normal controls. In particular, we have identified four plasma proteins—BSP, OMD, ACY1, and GHR—with consistent alterations in PD, one of which (GHR) also predicted subsequent cognitive decline in multiple cohorts, across multiple cognitive testing instruments. Our results open up new avenues for mechanistic investigation, suggesting that "near-proteomic" profiling of blood from individuals with PD may be a powerful approach both for the development of clinical tools and for insight into the pathophysiology of this currently incurable disease.

## Supporting information

S1 Fig QC measures. (A) Discovery Cohort Venn diagram shows the number of proteins that passed QC filters (968 proteins) and number of proteins that failed one or both of the QC criteria. Out of a total of 1,129 proteins, 161 were excluded because of high CVs (>20%) or high proportions (>25%) of measurements outside the assay’s limits of detection. (B) Replication Cohort Venn diagram shows that 33 proteins were removed because of CV greater than 20%, leaving a total of 1,272 proteins for downstream analyses. CV, coefficient of variation; QC, quality control.
(TIF)

S2 Fig Effect of levodopa therapy on plasma protein levels tested by paired permutation test. Volcano plot showing the effect of dopaminergic therapy on plasma protein levels, tested in 10 PD individuals, comparing ON versus OFF medication state in the same individuals. Nominally significant differences in ON versus OFF state were found for only one protein (paired permutation test nominal *p <* 0.01). PD, Parkinson’s disease.
(TIF)

S3 Fig Plasma levels of GHR and ACY1 predict cognitive decline in individuals with PD from Discovery Cohort. Differences in subsequent rates of clinical conversion to MCI or dementia in the Discovery Cohort stratified by GHR or ACY1 levels at baseline (shown as tertiles) are unaffected by education. (A,C,E) Cox regression curves showing adjusted trajectories for each tertile of baseline biomarker measures and (B,D,F) forest plots depicting hazard ratios for groups as defined by tertile of biomarker measures at baseline and covariates. (A,B) Results for ACY1 without adjusting for education. (C-F) Results for Cox proportional hazards analyses adjusting for education for GHR (C-D) and ACY1 (E-F), respectively. (G) Results from ANOVA (χ2 statistic, *p*-value) comparing Cox proportional hazards model with education (model 2) and without education (model 1). ACY1, aminoacylase-1; GHR, growth hormone receptor; MCI, mild cognitive impairment; PD, Parkinson’s disease.
(TIF)

S4 Fig Plasma levels of GHR predict cognitive decline in individuals with PD from Replication Cohort. Differences in subsequent rates of clinical conversion to MCI or dementia in the Replication (UTSW) Cohort stratified by GHR levels at baseline (shown as tertiles) are unaffected by education. (A) Cox regression curve showing adjusted trajectories for each tertile of baseline GHR measures and (B) forest plots depicting hazard ratios for groups as defined by GHR tertile, sex, age, disease duration, and the additional covariate of education. (C) Results from ANOVA (χ2 statistic and *p*-value) comparing Cox proportional hazards model with education (model 2) and without (model 1). GHR, growth hormone receptor; MCI, mild cognitive impairment; PD, Parkinson’s disease; UTSW, University of Texas Southwestern Medical Center.
(TIF)

S1 Table SOMAScan levels (log_10_ of RFU) of 1,129 proteins, and demographic data for 141 participants (96 PD and 45 NC) from Discovery (Udall) cohort. Proteins showing coefficient of variation greater than 20%, and more than 25% of measurements outside either the LLOD or ULOD were excluded form downstream analyses. Out of 1,129 proteins, 161 were excluded. LEDD, levodopa equivalent daily dose; LLOD, lower limit of detection; NC, neurologically normal control; PD, Parkinson’s disease; RFU, relative fluorescence unit; ULOD, upper limit of detection.
(XLSX)

S2 Table Candidate PD biomarkers from Discovery Cohort analysis (140 proteins). Proteins that differentiated PD and NC individuals (*p* < 0.005) were identified by applying a multivariate linear regression model to the Discovery Cohort data (96 PD and 45 NC). Protein names, ENTREZ symbols, *p*-value from regression model (differentiating PD versus NC status, adjusted for age at plasma sampling, sex, and LEDD), and direction of change in PD compared to NC are shown. *p*-Value from regression model differentiating PD and NC group adjusted for age at plasma collection, sex, and the LEDD. FDR = Benjamini-Hochberg adjusted *p*-value, 968 tests. FDR, false discovery rate; LEDD, levodopa equivalent daily dose; NC, neurologically normal control; PD, Parkinson’s disease.
(XLSX)

S3 Table SOMAScan levels (log_10_ of RFU) of 1,305 proteins and demographic data for 376 research participants (59 ALS, 215 PD, and 102 NC) from Replication Cohort. Three outlier individuals and 33 proteins showing coefficient of variation greater than 20% were excluded from analyses. ALS, amyotrophic lateral sclerosis; NC, neurologically normal control; PD, Parkinson’s disease; RFU, relative fluorescence unit.
(XLSX)

S4 Table Multiple regression FDR-adjusted *p*-values for top 10 proteins in Discovery and Replication Cohort. Top 10 plasma biomarker candidates (ranked by Stability Selection) that differentiated PD participants versus NC participants in the Discovery Cohort; four replicate associations in the Replication Cohort. Here, we report *p*-values from multiple linear regression model differentiating PD versus NC status, adjusted for age at plasma sampling, sex, and LEDD in Discovery Cohort. In the Replication Cohort, the effect of treatment group (PD versus NC) was adjusted for age at plasma sampling, sex, clinical site, and batch effect. Adjustment for the FDR was performed using the Benjamini-Hochberg method. FDR, false discovery rate; LEDD, levodopa equivalent daily dose; NC, neurologically normal control; PD, Parkinson’s disease.
(DOCX)

S5 Table Change in protein levels in ON versus OFF dopaminergic state, and after systematic perturbation of samples of extra freeze–thaw and prolonged (30 minutes) room temperature exposure. For ON versus OFF analysis, plasma samples were collected during ON–OFF state for each of the 10 PD participants (5 females and 5 males of age >50 years) randomly selected from the BioFIND database. Percentage of change of raw RFUs and log_10_(RFU) differences between ON and OFF state are shown for 1,272 proteins. Only two proteins (IGFBP-1 and PCSK9) significantly differentiated ON versus OFF dopaminergic state. **Paired *t* test nominal *p*-value < 0.01, two-tailed. IGFBP-1, insulin-like growth factor binding protein 1; PCSK9, proprotein convertase subtilisin/kexin type 9; PD, Parkinson’s disease; RFU, relative fluorescence unit.
(XLSX)

S6 Table Results from mixed-effects linear models in Discovery Cohort. For our first model (model 1), the response variable DRS was modeled as a fixed effect of time–protein interaction, age, sex, disease duration, and baseline DRS and as a random effect of participant. Model 2 adjusts for education by including it as an additional fixed effect. Shown are time–protein interaction coefficients (*β*_*time*∙*prot*_) and their corresponding FDR-adjusted *p*-values for model 1 and model 2. DRS, Mattis Dementia Rating Scale-2; FDR, false discovery rate.
(DOCX)

S1 STROBE Checklist STROBE, Strengthening the Reporting of Observations Studies in Epidemiology. (DOCX)

S1 Analysis Plan (DOCX)

## Abbreviations

ACY1: aminoacylase-1
AD: Alzheimer’s disease
ALS: amyotrophic lateral sclerosis
BSP: bone sialoprotein
CSF: cerebrospinal fluid
CTCC: Clinical Trials Coordination Center
CV: coefficient of variation
DRS: Mattis Dementia Rating Scale-2
FDA: US Food and Drug Administration
FDR: false discovery rate
GH: growth hormone
GHR: growth hormone receptor
HR: hazard ratio
HSD: honest significant difference
IGF-1: insulin-like growth factor 1
IGFBP-1: insulin-like growth factor binding protein 1
IRB: institutional review board
LASSO: least absolute shrinkage and selection operator
LEDD: levodopa equivalent daily dose
LLOD: lower limit of detection
MCI: mild cognitive impairment
MoCA: Montreal Cognitive Assessment
NC: neurologically normal control
OMD: osteomodulin
PCSK9: proprotein convertase subtilisin/kexin type 9
PD: Parkinson’s disease
PDBP: Parkinson’s Disease Biomarker Program
QC: quality control
RFU: relative fluorescence unit
SD: standard deviation
SOMAmer: slow off-rate modified aptamer
STROBE: Strengthening the Reporting of Observations Studies in Epidemiology
ULOD: upper limit of detection
UPenn: University of Pennsylvania
UTSW: University of Texas Southwestern Medical Center
