# Supplementary figures and images for: Characterization of Parkinson’s disease using blood-based biomarkers: A multicohort proteomic analysis
Source: PLoS Med. 2019 Oct 11;16(10):e1002931. doi: 10.1371/journal.pmed.1002931 (PMC6788685; doi:10.1371/journal.pmed.1002931)

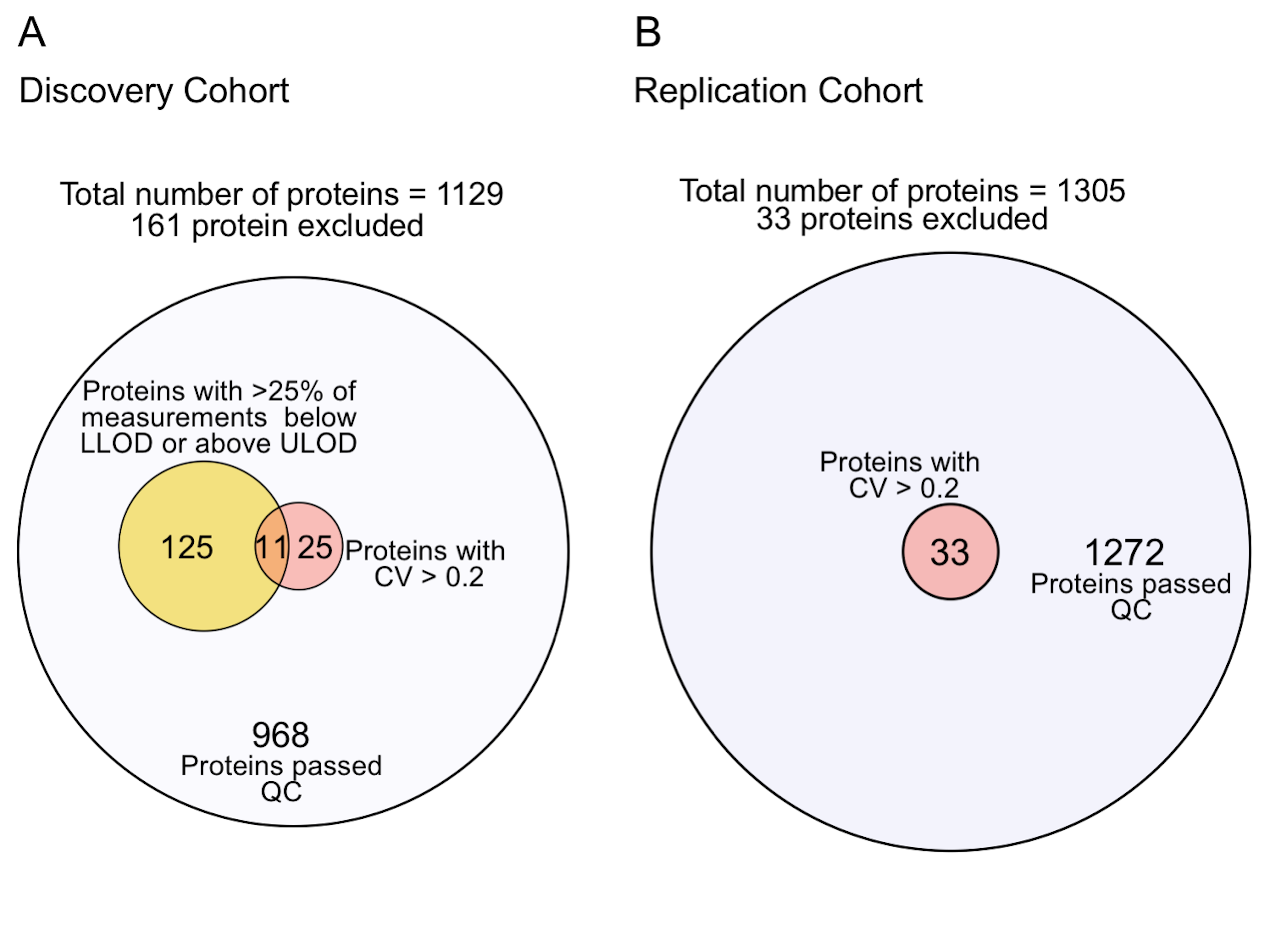

Supplement: S1 Fig — (A) Discovery Cohort Venn diagram shows the number of proteins that passed QC filters (968 proteins) and number of proteins that failed one or both of the QC criteria. Out of a total of 1,129 proteins, 161 were excluded because of high CVs (>20%) or high proportions (>25%) of measurements outside the assay’s limits of detection. (B) Replication Cohort Venn diagram shows that 33 proteins were removed because of CV greater than 20%, leaving a total of 1,272 proteins for downstream analyses. CV, coefficient of variation; QC, quality control. (TIF) [file pmed.1002931.s001.tif]

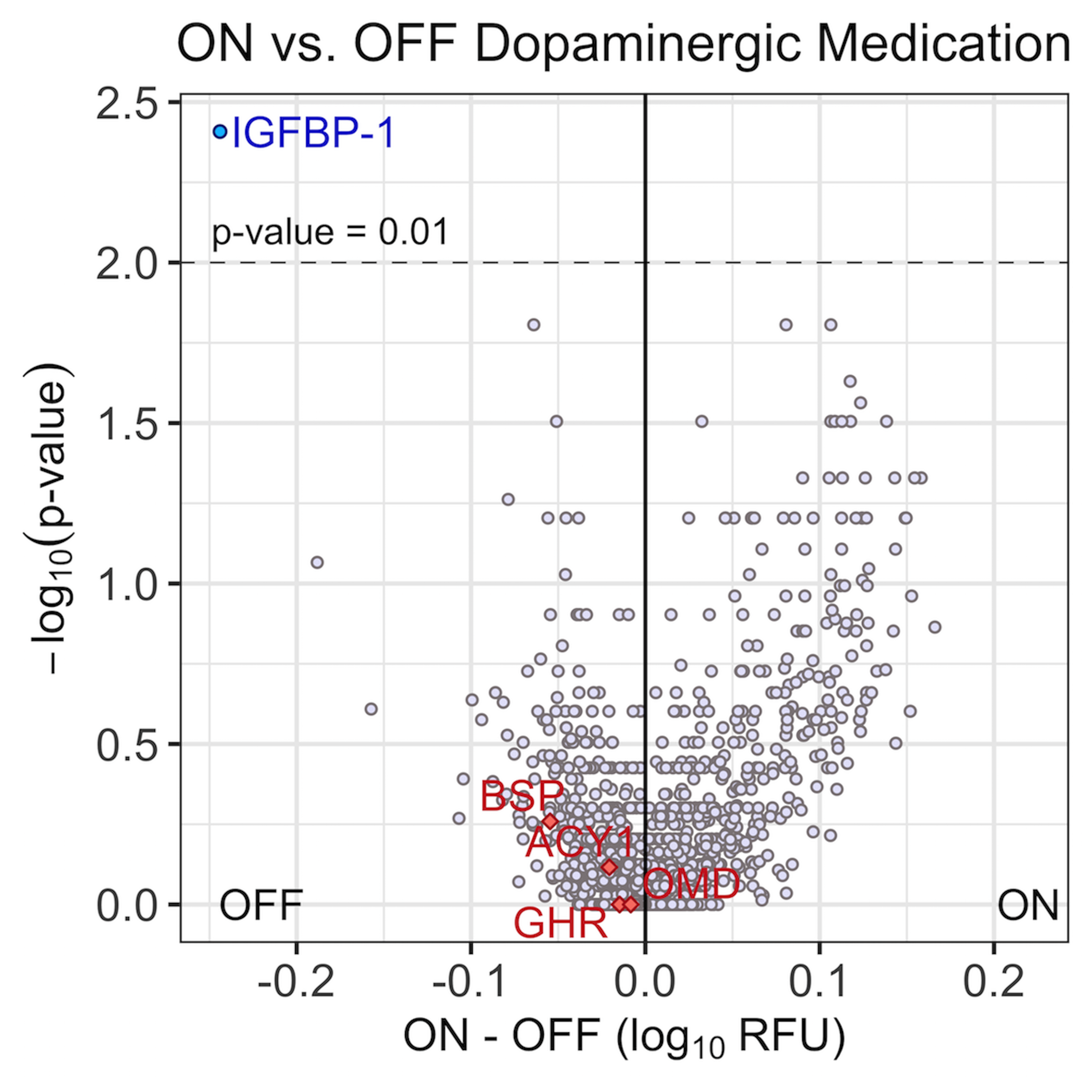

Supplement: S2 Fig — Volcano plot showing the effect of dopaminergic therapy on plasma protein levels, tested in 10 PD individuals, comparing ON versus OFF medication state in the same individuals. Nominally significant differences in ON versus OFF state were found for only one protein (paired permutation test nominal p < 0.01). PD, Parkinson’s disease. (TIF) [file pmed.1002931.s002.tif]

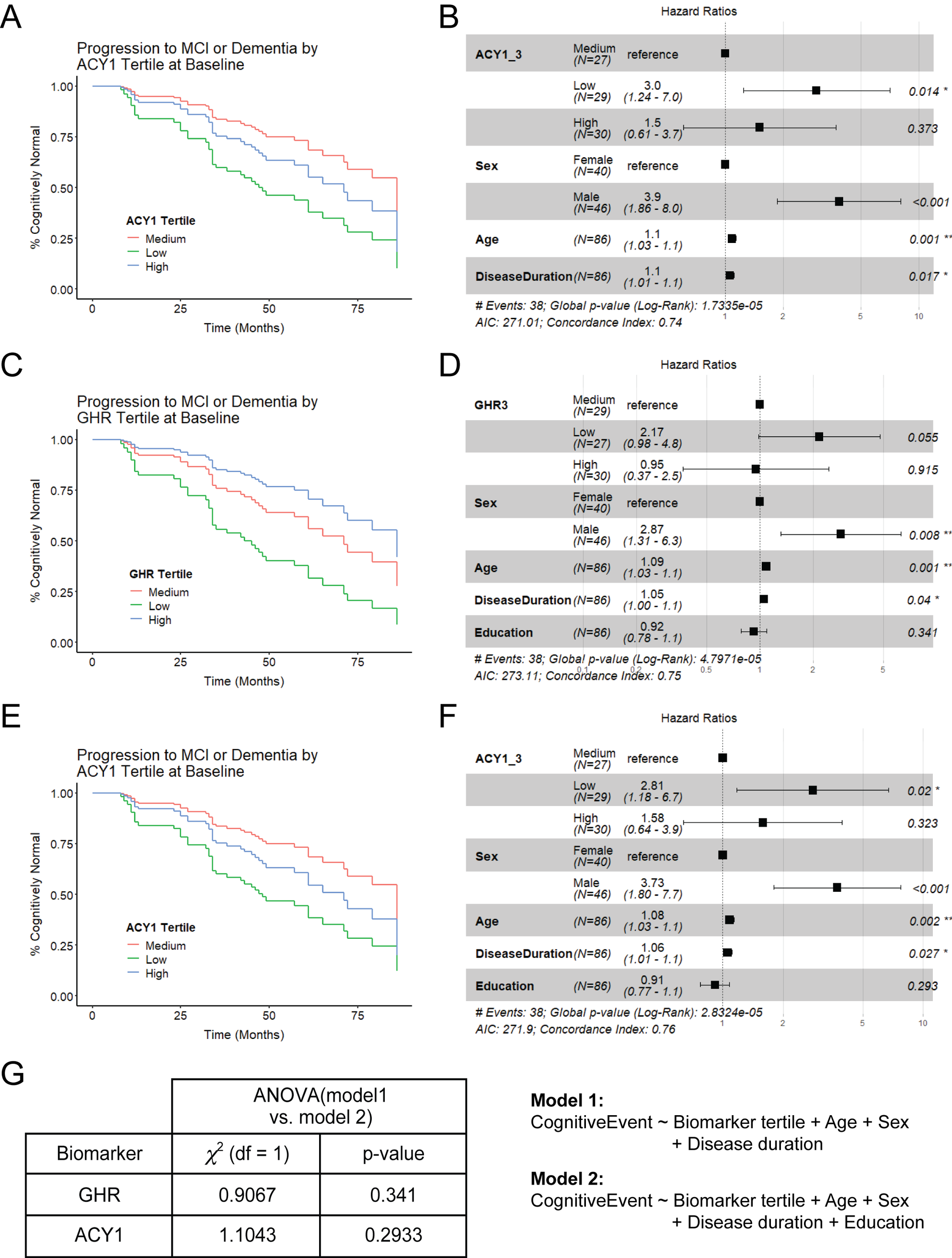

Supplement: S3 Fig — Differences in subsequent rates of clinical conversion to MCI or dementia in the Discovery Cohort stratified by GHR or ACY1 levels at baseline (shown as tertiles) are unaffected by education. (A,C,E) Cox regression curves showing adjusted trajectories for each tertile of baseline biomarker measures and (B,D,F) forest plots depicting hazard ratios for groups as defined by tertile of biomarker measures at baseline and covariates. (A,B) Results for ACY1 without adjusting for education. (C-F) Results for Cox proportional hazards analyses adjusting for education for GHR (C-D) and ACY1 (E-F), respectively. (G) Results from ANOVA (χ2 statistic, p-value) comparing Cox proportional hazards model with education (model 2) and without education (model 1). ACY1, aminoacylase-1; GHR, growth hormone receptor; MCI, mild cognitive impairment; PD, Parkinson’s disease. (TIF) [file pmed.1002931.s003.tif]

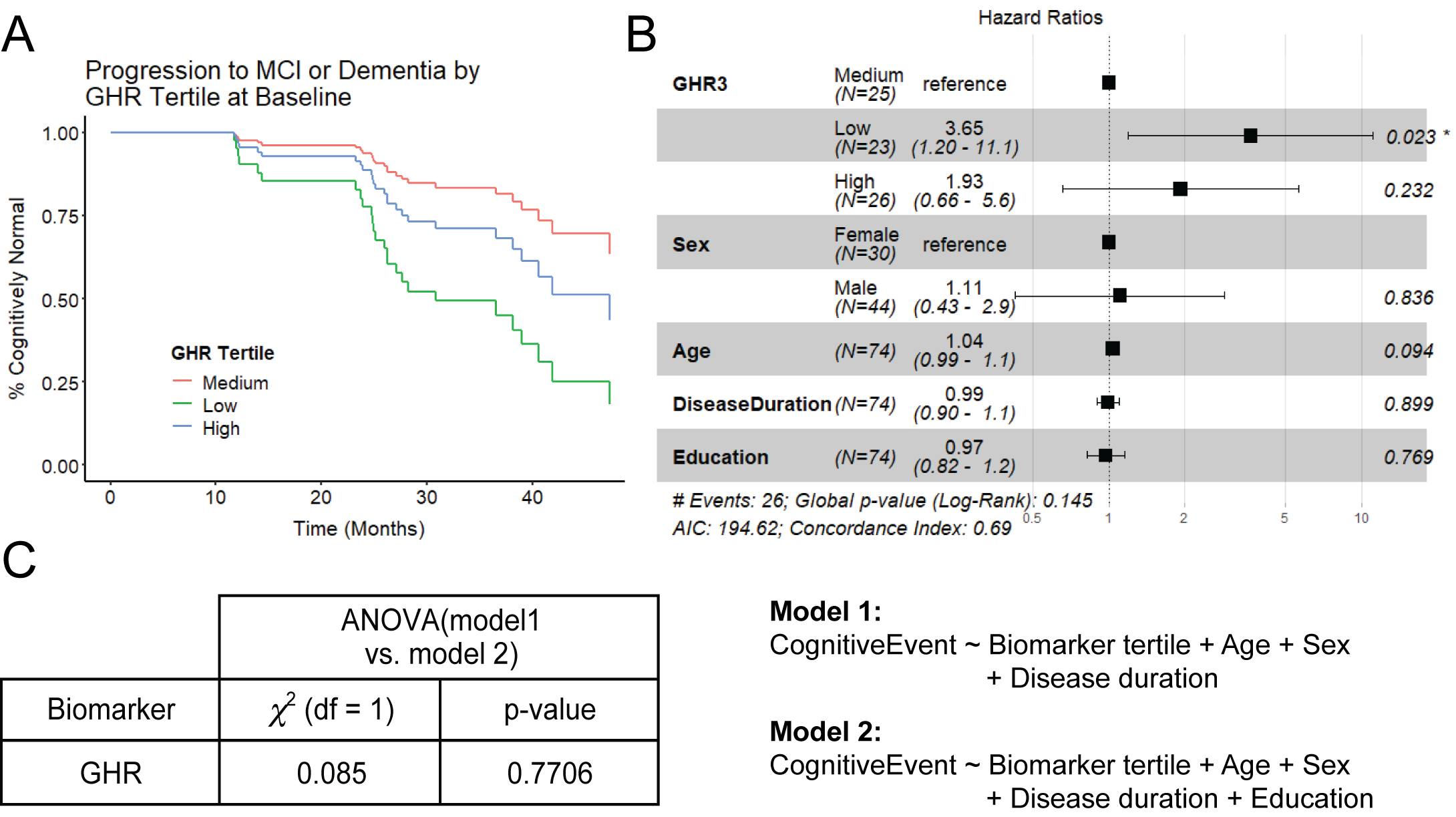

Supplement: S4 Fig — Differences in subsequent rates of clinical conversion to MCI or dementia in the Replication (UTSW) Cohort stratified by GHR levels at baseline (shown as tertiles) are unaffected by education. (A) Cox regression curve showing adjusted trajectories for each tertile of baseline GHR measures and (B) forest plots depicting hazard ratios for groups as defined by GHR tertile, sex, age, disease duration, and the additional covariate of education. (C) Results from ANOVA (χ2 statistic and p-value) comparing Cox proportional hazards model with education (model 2) and without (model 1). GHR, growth hormone receptor; MCI, mild cognitive impairment; PD, Parkinson’s disease; UTSW, University of Texas Southwestern Medical Center. (TIF) [file pmed.1002931.s004.tif]
