# Supplementary material for: Characterization of Parkinson’s disease using blood-based biomarkers: A multicohort proteomic analysis
Source: PLoS Med. 2019 Oct 11;16(10):e1002931. doi: 10.1371/journal.pmed.1002931 (PMC6788685; doi:10.1371/journal.pmed.1002931)
Supplement: S4 Table — Top 10 plasma biomarker candidates (ranked by Stability Selection) that differentiated PD participants versus NC participants in the Discovery Cohort; four replicate associations in the Replication Cohort. Here, we report p-values from multiple linear regression model differentiating PD versus NC status, adjusted for age at plasma sampling, sex, and LEDD in Discovery Cohort. In the Replication Cohort, the effect of treatment group (PD versus NC) was adjusted for age at plasma sampling, sex, clinical site, and batch effect. Adjustment for the FDR was performed using the Benjamini-Hochberg method. FDR, false discovery rate; LEDD, levodopa equivalent daily dose; NC, neurologically normal control; PD, Parkinson’s disease. (DOCX) [file pmed.1002931.s008.docx]

**S4 Table.** **Multiple regression FDR adjusted p-values for top 10 proteins in Discovery and Replicate Cohort**. Top 10 plasma biomarker candidates (ranked by Stability Selection) that differentiated Parkinson’s Disease (PD) patients *versus* neurologically normal (NC) subjects in the Discovery Cohort; 4 replicate associations in the Replication Cohort. Here we report p-values from multiple linear regression model differentiating PD *vs*. NC status, adjusted for age at plasma sampling, sex and LEDD in Discovery Cohort. In the Replication Cohort the effect of treatment group (PD *vs*. NC) was adjusted for age at plasma sampling, sex, clinical site, and batch effect. Adjustment for the False Discovery Rate (FDR) was performed using the Benjamini-Hochberg method.

|  | | Discovery Cohort | | | Replication Cohort | |
| --- | --- | --- | --- | --- | --- | --- |
| *Protein Name* | *Entrez Symbol* | *Nominal*  *p-value* | *FDR p-value*  *968 tests* | *Nominal*  *p-value* | | *FDR p-value*  *10 tests* |
| Aminoacylase-1 | ACY1 | 1.11E-04 | 1.86E-03 | 8.70E-03 | | 2.18E-02 |
| BDNF | BDNF | 7.22E-04 | 7.81E-03 | 2.98E-01 | | 4.26E-01 |
| BSP | IBSP | 3.88E-03 | 2.82E-02 | 2.06E-05 | | 1.03E-04 |
| C1r | C1R | 6.30E-07 | 6.10E-05 | 1.99E-01 | | 3.98E-01 |
| Growth hormone receptor | GHR | 1.01E-05 | 3.49E-04 | 8.92E-04 | | 2.97E-03 |
| IFN-g R1 | IFNGR1 | 4.64E-03 | 3.23E-02 | 3.69E-01 | | 4.61E-01 |
| OMD | OMD | 2.58E-03 | 2.14E-02 | 9.14E-06 | | 9.14E-05 |
| RAN | RAN | 5.30E-08 | 1.28E-05 | 8.78E-01 | | 8.78E-01 |
| SRCN1 | SRC | 3.31E-06 | 2.00E-04 | 2.79E-01 | | 4.65E-01 |
| tau | MAPT | 4.57E-04 | 5.26E-03 | 4.32E-01 | | 4.79E-01 |
