# Supplementary material for: Characterization of Parkinson’s disease using blood-based biomarkers: A multicohort proteomic analysis
Source: PLoS Med. 2019 Oct 11;16(10):e1002931. doi: 10.1371/journal.pmed.1002931 (PMC6788685; doi:10.1371/journal.pmed.1002931)
Supplement: S6 Table — For our first model (model 1), the response variable DRS was modeled as a fixed effect of time–protein interaction, age, sex, disease duration, and baseline DRS and as a random effect of participant. Model 2 adjusts for education by including it as an additional fixed effect. Shown are time–protein interaction coefficients (βtime∙prot) and their corresponding FDR-adjusted p-values for model 1 and model 2. DRS, Mattis Dementia Rating Scale-2; FDR, false discovery rate. (DOCX) [file pmed.1002931.s010.docx]

**S6 Table**. **Results from mixed-effects linear models in Discovery Cohort**. For our first model (model 1), the response variable dementia rating scale-2 (DRS) was modeled as a fixed effect of time-protein interaction, age, sex, disease duration, and baseline DRS, and a random effect of patient. Model 2 adjusts for education by including it as an additional fixed effect. Shown are time-protein interaction coefficients ($\beta_{time\cdot prot}$) and their corresponding FDR-adjusted p-values for model 1 and model 2.

|  | Model 1 (no education) | | Model 2 (with education) | |
| --- | --- | --- | --- | --- |
| Protein | $\beta_{time\cdot prot}$ | FDR-adj. p-value | $\beta_{time\cdot prot}$ | FDR-adj. p-value |
| Growth hormone  receptor | 0.09051 | 8.72E-06 | 0.09050 | 8.73E-06 |
| Aminoacylase-1 | 0.04779 | 2.57E-02 | 0.04764 | 2.33E-02 |
| OMD | -0.04574 | 2.57E-02 | -0.04644 | 2.33E-02 |
| BSP | -0.02881 | 1.26E-01 | -0.03013 | 1.10E-01 |
