# Supplementary material for: Characterization of Parkinson’s disease using blood-based biomarkers: A multicohort proteomic analysis
Source: PLoS Med. 2019 Oct 11;16(10):e1002931. doi: 10.1371/journal.pmed.1002931 (PMC6788685; doi:10.1371/journal.pmed.1002931)
Supplement: S1 STROBE Checklist — (DOCX) [file pmed.1002931.s011.docx]

STROBE Statement—Checklist of items that should be included in reports of ***cohort studies***

|  | Item No | Recommendation |
| --- | --- | --- |
| **Title and abstract** | 1 | (*a*) Indicate the study’s design with a commonly used term in the title or the abstract  **Abstract: Methods and Findings, paragraph 1** |
|  |  | (*b*) Provide in the abstract an informative and balanced summary of what was done and what was found  **Abstract: Methods and Findings, paragraphs 1-2** |
| Introduction | | |
| Background/rationale | 2 | Explain the scientific background and rationale for the investigation being reported  **Introduction; paragraphs 1-2** |
| Objectives | 3 | State specific objectives, including any prespecified hypotheses  **Introduction: paragraph 2** |
| Methods | | |
| Study design | 4 | Present key elements of study design early in the paper  **Methods: paragraph 1, and see also Fig 1** |
| Setting | 5 | Describe the setting, locations, and relevant dates, including periods of recruitment, exposure, follow-up, and data collection  **Methods - Cohorts and sample collection; Protein Quantification** |
| Participants | 6 | (*a*) Give the eligibility criteria, and the sources and methods of selection of participants. Describe methods of follow-up  **Methods - Cohorts and sample collection: UPenn Udall Discovery Cohort, Multi-site PDBP Replication Cohort, BioFIND cohort samples** |
|  |  | (*b*) For matched studies, give matching criteria and number of exposed and unexposed  **N/A** |
| Variables | 7 | Clearly define all outcomes, exposures, predictors, potential confounders, and effect modifiers. Give diagnostic criteria, if applicable  **Methods - Data pre-processing and Statistical Analyses: PD Progression Analysis** |
| Data sources/ measurement | 8* | For each variable of interest, give sources of data and details of methods of assessment (measurement). Describe comparability of assessment methods if there is more than one group  **Methods - Pre-processing and QC od SOMAScan protein data, Survival analysis** |
| Bias | 9 | Describe any efforts to address potential sources of bias  **Methods - Pre-processing and QC of SOMAScan protein data, Data Processing and Statistical Analyses: Hierarchical clustering, Stability Selection Ranking, Testing the Effect of Levodopa Therapy** |
| Study size | 10 | Explain how the study size was arrived at  **Discovery Cohort used a convenience sample. For the Replication Cohort, we used all individuals with at least 1 year of longitudinal follow-up data from the PDBP at the time of biomarker screening.** |
| Quantitative variables | 11 | Explain how quantitative variables were handled in the analyses. If applicable, describe which groupings were chosen and why  **Methods: Pre-processing and QC of SOMAScan protein data, PD progression Analysis** |
| Statistical methods | 12 | (*a*) Describe all statistical methods, including those used to control for confounding  **Methods: Data Processing and Statistical Analysis** |
|  |  | (*b*) Describe any methods used to examine subgroups and interactions  **Methods - Data Processing and Statistical Analyses - PD Progression Analysis: Linear mixed-effect model analysis wit time-by-protein interaction** |
|  |  | (*c*) Explain how missing data were addressed  **Only complete cases were analysed** |
|  |  | (*d*) If applicable, explain how loss to follow-up was addressed  **N/A** |
|  |  | (*e*) Describe any sensitivity analyses  **N/A** |
| Results | | |
| Participants | 13* | (a) Report numbers of individuals at each stage of study—eg numbers potentially eligible, examined for eligibility, confirmed eligible, included in the study, completing follow-up, and analysed  **Table 1, Results: Replication of biomarker associations with PD in PDBP Cohort, paragraph1 and Biomarker measures in ALS, a neurodegenerative disease with motor and cognitive features** |
|  |  | (b) Give reasons for non-participation at each stage  **N/A** |
|  |  | (c) Consider use of a flow diagram  **N/A** |
| Descriptive data | 14* | (a) Give characteristics of study participants (eg demographic, clinical, social) and information on exposures and potential confounders  **Table 1** |
|  |  | (b) Indicate number of participants with missing data for each variable of interest  **N/A (complete case analysis)** |
|  |  | (c) Summarise follow-up time (eg, average and total amount)  **Results: GHR, ACY1, and OMD as predictors of cognitive decline** |
| Outcome data | 15* | Report numbers of outcome events or summary measures over time  **N/A** |
| Main results | 16 | (*a*) Give unadjusted estimates and, if applicable, confounder-adjusted estimates and their precision (eg, 95% confidence interval). Make clear which confounders were adjusted for and why they were included  **Supplementary Tables, S2, S4** and **S6** |
|  |  | (*b*) Report category boundaries when continuous variables were categorized  **Methods: Survival analysis** |
|  |  | (*c*) If relevant, consider translating estimates of relative risk into absolute risk for a meaningful time period  **N/A** |
| Other analyses | 17 | Report other analyses done—eg analyses of subgroups and interactions, and sensitivity analyses  **Methods: Stability Selection Ranking** |
| Discussion | | |
| Key results | 18 | Summarise key results with reference to study objectives  **Discussion: paragraph 1** |
| Limitations | 19 | Discuss limitations of the study, taking into account sources of potential bias or imprecision. Discuss both direction and magnitude of any potential bias  **Discussion: paragraph 4** |
| Interpretation | 20 | Give a cautious overall interpretation of results considering objectives, limitations, multiplicity of analyses, results from similar studies, and other relevant evidence  **Discussion: paragraph 2** |
| Generalisability | 21 | Discuss the generalisability (external validity) of the study results  **Discussion: paragraph 2 and 5** |
| Other information | | |
| Funding | 22 | Give the source of funding and the role of the funders for the present study and, if applicable, for the original study on which the present article is based  **This study was funded by the NIH (NINDS P50 NS053488, UO1 NS082134 and UO1 NS097056). Alice Chen-Plotkin holds the Parker Family endowed professorship and has also been supported by the Benaroya Fund. None of the funders had any role in study design or interpretation of results.** |

*Give information separately for exposed and unexposed groups.

**Note:** An Explanation and Elaboration article discusses each checklist item and gives methodological background and published examples of transparent reporting. The STROBE checklist is best used in conjunction with this article (freely available on the Web sites of PLoS Medicine at http://www.plosmedicine.org/, Annals of Internal Medicine at http://www.annals.org/, and Epidemiology at http://www.epidem.com/). Information on the STROBE Initiative is available at http://www.strobe-statement.org.
