# Supplementary material for: Characterization of Parkinson’s disease using blood-based biomarkers: A multicohort proteomic analysis
Source: PLoS Med. 2019 Oct 11;16(10):e1002931. doi: 10.1371/journal.pmed.1002931 (PMC6788685; doi:10.1371/journal.pmed.1002931)
Supplement: S1 Analysis Plan — (DOCX) [file pmed.1002931.s012.docx]

**Analysis Plan, Posavi *et al.***

1. This study had a Training Set/Test Set design. Both sets of participants would be pre-designated (*i.e.* individuals from the Test Set could not be re-grouped into the Training Set and vice versa), with the Training Set consisting of PD and control participants recruited from the University of Pennsylvania (single site), and the Test Set consisting of PD and control participants recruited from the Parkinson’s Disease Biomarker Program (PDBP) cohort.
2. Selection of University of Pennsylvania participants was based on diagnosis, with care taken to ensure that PD and control groups were balanced in age and sex.
3. The final selection of PDBP cohort participants was made based on consultation with the PDBP staff to identify individuals from clinical sites with the longest follow-up duration at the time of sampling. In addition, PDBP staff helped us to ensure that PD and control groups were balanced in age and sex, and that samples were allotted to batches of the testing platform without skews in age, sex, diagnosis, or clinical site.
4. In the Training Set, linear regression models would be used to predict plasma protein concentrations of each potential biomarker based on disease vs. control category, adjusted for the covariates age, sex, and levodopa equivalent daily dose (LEDD), nominating all biomarkers that met a nominal p-value threshold of p<0.005 for disease vs. control effect. In response to reviewer comments, we subsequently added correction for multiple hypothesis testing at this stage using the Benjamini-Hochberg method. We had not pre-specified the correction for multiple hypothesis testing at the Training Set stage since our goal was to cast a wide net for potential biomarkers to enter the next step, Stability Selection.
5. Markers derived from the Training Set would be ranked by Stability Selection – the number of proteins and individuals left out of the bag, as well as the number of iterations used, would be tuned to maximize model performance within the Training Set. Ranking of biomarkers by Stability Selection would be based on the number of iterations in which LASSO reported a non-zero coefficient (*i.e.* the given protein was informative within the model).
6. Based on findings from Stability Selection, a smaller set (in the end, the top 10 biomarkers) of biomarkers would be evaluated in the Test Set. The original plan was to use the exact same regression model in the Test Set, with the addition of covariates of site and batch if these effects were found to be significant in the Test Set. The analysis differed from the original plan in that we were unable to include LEDD in the model for the Test Set as this information was not universally available for PDBP cohort participants. However, all other aspects of the model remained the same.
